# Supplementary material for: Analysis of Sequential Micromixing Driven by Sinusoidally Shaped Induced-Charge Electroosmotic Flow
Source: Micromachines (Basel). 2022 Nov 16;13(11):1985. doi: 10.3390/mi13111985 (PMC9695967; doi:10.3390/mi13111985)
Supplement: Supplementary file 1 [file micromachines-13-01985-s001.zip › micromachines-2014567-supplementary.pdf]

Analysis of Sequential Micromixing Driven by Sinusoidally Shaped Induced-Charge Electroosmotic flow

Haizhen Sun[1, 3], Ziyi Li[1,3], Yongji Wu[1, 3], Xinjian Fan[1, 3, *], Minglu Zhu[1, 3, *], Tao Chen[2, 3] and Lining Sun[1, 3],

[1] School of Mechanical and Electric Engineering, Soochow University, Suzhou 215299, China

[2] School of Future Science and Engineering, Soochow University, Suzhou 215299, China

[3] Jiangsu Provincial Key Laboratory of Advanced Robotics, School of Mechanical and Electric Engineering, Soochow University, Suzhou 215123, China

[*] Correspondence: chent@suda.edu.cn (T. Chen); xinjianfan@suda.edu.cn (X. Fan)

Electronic Supporting Information (ESI)

Section S1. The 3D model and the boundary conditions used in the numerical simulation

A 3-D model of the same size as the designed micromixer was established. The governing equations and boundary conditions that are based on the 3-D geometric model are shown in Figure S1 and Table S1.

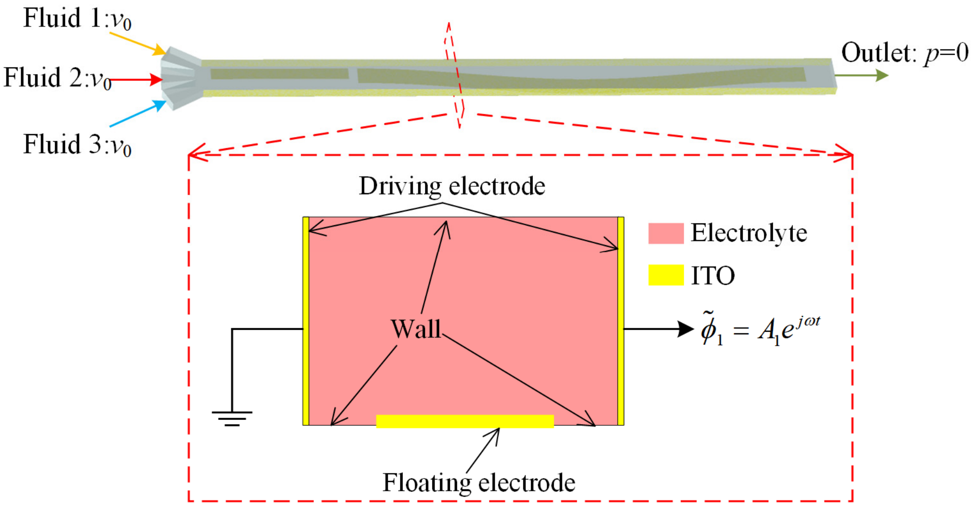

Figure S1 The 3D model and the boundary conditions used in the numerical simulation.

Table S1. Governing equations and boundary conditions employed in the numerical simulation

Position	Condition in electric field	Condition in flow field
Electrolyte	$\nabla^2\tilde{\varphi} = 0$	$-\nabla p + \nabla \cdot \left(\eta(\nabla u + (\nabla u)^T)\right) = 0, \nabla \cdot u = 0$
Driving electrodes	$\sigma(n \cdot \nabla\tilde{\varphi}) = j\omega C_0(\tilde{\varphi} - A)$	$\langle u_{slip}\rangle = \frac{\varepsilon}{2(1+\delta)\eta} Re((\tilde{\varphi} - A)(\tilde{E} - \tilde{E} \cdot n \cdot n)^*)$
Floating electrode	$\sigma(n \cdot \nabla\tilde{\varphi}) = j\omega C_0(\tilde{\varphi} - \varphi_0)$	$\langle u_{slip}\rangle = \frac{\varepsilon}{2(1+\delta)\eta} Re((\tilde{\varphi} - \varphi_0)(\tilde{E} - \tilde{E} \cdot n \cdot n)^*)$
Wall	$n \cdot \nabla\tilde{\varphi} = 0$	$u = 0$

Notes: $\tilde{\varphi} = Ae^{j\theta}$ is complex phasor amplitude of electrostatic potential $\varphi(\mathrm{t}) = \mathrm{A}\cos(\omega t + \theta)$ (j=$\sqrt{-1}$) and $\omega=2\pi f$ represents the angular frequency. p, η and u denote the flow pressure; the dynamic viscosity of the fluids and the flow velocity, respectively. $\tilde{E}$ represents the electric field phasor and ε is the fluidic permittivity; $\delta = C_d/C_s$ (C_d and C_s represent the capacitance of diffuse layer and the capacitance of stern layer, respectively). $\langle u_{slip} \rangle$ denotes the time-averaged induced-charge electroosmotic (ICEO) slip velocity. $Re(\cdots)$ indicates the real part of $(\cdots)$; n is the unit normal vector pointing into fluid.[1]

REFERENCES

(1) Bazant, M. Z.; Squires, T. M. Induced-charge electrokinetic phenomena: theory and microfluidic applications. Physical review letters 2004, 92 (6), 066101.
